# In Vitro Antitumor Effect of Oils Rich in CBD and THC Cannabis Extract in Canine Prostate Carcinoma Cell Lines

**DOI:** 10.3390/vetsci11100501

**Published:** 2024-10-13

**Authors:** Luís Gustavo Ramos de Moraes Calheiros, Giovana Pedro, Thayna Oliveira da Silva, Rogério Martins Amorim, Carlos Eduardo Fonseca Alves, Renée Laufer-Amorim

**Affiliations:** School of Veterinary Medicine and Animal Science, São Paulo State University (UNESP), Botucatu 18618-681, Brazil; lgrm.calheiros@unesp.br (L.G.R.d.M.C.); giovana.pedro@unesp.br (G.P.); thayna.o.silva@unesp.br (T.O.d.S.); rogerio.amorim@unesp.br (R.M.A.); carlos.e.alves@unesp.br (C.E.F.A.)

**Keywords:** comparative oncology, natural compounds, translational medicine, medicinal plants

## Abstract

**Simple Summary:**

Prostate cancer is one of the leading causes of cancer deaths worldwide, even when found early in the disease, in both humans and dogs. Prostate cancer in dogs is common and is very similar to human prostate cancer, making them excellent models for comparative studies. Cannabidiol and Δ9-tetrahydrocannabinol are the two main components of *Cannabis sativa* and have been shown to have anti-cancer properties. In this study, extracts rich in cannabidiol or Δ9-tetrahydrocannabinol inhibited the growth of two canine prostate carcinoma cell lines. These results provide new information about the use of these natural compounds in canine models, which gives us the opportunity for further studies, both in the laboratory and in animals, to discover how these compounds act in the body, using dogs as a natural model for prostate carcinoma.

**Abstract:**

Prostate cancer is one of the leading causes of cancer-related deaths worldwide, even when diagnosed at an early stage in humans and dogs. Dogs have a significant incidence of spontaneous prostate cancer, which is highly similar to human androgen-independent prostate cancer and represents a valuable model for comparative studies. Cannabidiol (CBD) and Δ9-tetrahydrocannabinol (THC) are the two main cannabinoids extracted from *Cannabis sativa* and have demonstrated antiproliferative and anti-invasive properties in different tumor types. In this study, CBD or THC-rich extracts inhibited the proliferation of two canine prostatic carcinoma cell lines, PC1 and PC2, showing an IC50 of 3.43 and 3.57 μM for CBD rich extracts, and 4.90 and 4.48 μM THC rich extracts, respectively. Cell death was also observed with both Annexin V and Propidium iodide staining for the canine cell lines. These results provide new information concerning the use of rich oil in canine PC and open a promising opportunity for further in vitro and in vivo studies to establish the mechanisms of action of these compounds using dogs as a natural model for prostatic carcinoma.

## 1. Introduction

Prostate cancer (PC) is one of the main causes of cancer-related death worldwide, affecting men between 45 and 60 years of age, and is the most commonly diagnosed non-skin tumor among male patients [1,2]. Even when diagnosed early, the therapeutic risk-benefit ratio is uncertain, with radical treatments such as prostatectomy being associated with high cure rates but a large recurrence rate within 10 years [1,3]. Patients with metastatic PC usually have castration-resistant prostate cancer (CRPC), which does not respond to androgen privation therapy and is the main cause of mortality and morbidity associated with PC [3].

Dogs are the only large mammals that spontaneously develop PC, and are considered a good natural model for CRPC, even in intact animals, since most canine PCs are androgen-negative, and they share the same environment as men, representing a valuable model for studying disease progression [4,5,6]. These findings stand out from murine models because they do not present limitations such as anatomy, life expectancy, and body weight that affect the carcinogenesis process [4,5], thus providing an excellent therapeutic model that can predict human outcomes [6].

In dogs, the prevalence of PC is 2–12%, and PC is more frequent in animals over seven years old, intact or spayed, with an undifferentiated morphology, aggressive nature, and metastatic rates reaching 80%, mainly in the bones, regional lymph nodes, lungs, and liver [6,7,8]. Unlike in humans, it is diagnosed later in dogs due to the inefficiency of diagnostic screening tests, such as PSA, in dogs. Canine PC has a poor prognosis due to its aggressive local and systemic behavior, low survival time (one month), and poor quality of life due to the absence of effective therapeutic protocols [6,7,9].

Canine PCs commonly exhibit spontaneous metastasis, loss of androgen receptor (AR) expression, and the expression of proteins such as NKX3.1 and PTEN, which are associated with aggressive tumor behaviors in dogs and are also found in CRPC. These targets have great potential for investigating tumor progression mechanisms, metastasis, and possible diagnostic and prognostic markers at the molecular level [5,8].

In men, the available treatments are expensive and have serious side effects, such as not being curative and leading to a resistant disease phenotype [2]. To date, no drugs are available to treat CRPC, and it is considered an incurable disease. However, combined therapies have proven effective in treating CRPC and may be useful in prolonging patients’ life expectancy [2]. Therefore, dogs with CRPC could be an interesting model for studying new treatment protocols [4].

Medicinal plants have recently attracted increased attention, playing an important role in traditional medicine research for cancer treatment to minimize side effects or as an adjuvant treatment to conventional therapies [2]. Plant-derived compounds have already been widely used due to their anti-inflammatory, antimicrobial, and antitumoral properties, in addition to restoring chemosensitivity in resistant tumor cells. Several currently used anticancer agents, such as paclitaxel, taxol, docetaxel, vincristine, and vinblastine, are derived from plants [2].

*Cannabis sativa* has been used medicinally for thousands of years, and the plant contains more than 550 chemical compounds, with 100 phytocannabinoids already identified [10]. Cannabidiol (CBD) and Δ9-tetrahydrocannabinol (THC) are the two main cannabinoids extracted from C. sativa and have demonstrated great pharmacological activity in various diseases, as well as antiproliferative and anti-invasive properties in different neoplasm types [11,12,13].

CBD inhibits the proliferation of tumor cells in vitro, and several studies have shown that CBD has antiproliferative and proapoptotic effects and prevents cell migration, adhesion, and invasion [11,14]. THC has been shown to exert antiproliferative and proapoptotic effects on different cancer types, such as human lung, glioma, thyroid, lymphoma, pancreas, uterus, breast, prostate, and colorectal carcinoma, in both in vitro and in vivo studies [11,14]. In addition, THC has been shown to have tumor selectivity, inhibiting tumor cell growth without affecting the growth of corresponding non tumor cells [12].

Tumor cell migration followed by invasion at secondary sites is the main metastasis mechanism and remains one of the main obstacles in cancer treatment, and in addition to preventing cell proliferation and inhibiting neovascularization, migration, and adhesion [12,14], cannabinoids also prevent epithelial–mesenchymal transition, one of the pivotal points in the metastatic cascade [13]. There are few studies concerning the use of C. sativa derivatives as anticancer agents. The proliferation of five different canine tumor cell lines was reduced after treatment with a combination of vincristine and CBD in a synergistic mode of action [14]. Human and canine glioma cell lines are sensitive to CBD treatment [15]. Another in vitro comparative study with human and canine lymphoma cell lines demonstrated an anticancer effect of CBD and THC. One canine T-cell lymphoma cell line (TCL-CL1) was sensitive only to CBD [16].

The aim of this work was to test the antitumor effects of rich CBD and THC extract oils on canine.

## 2. Materials and Methods

Antibiotic antimycotic solution—Gibco™, Thermo Fisher Scientific, Waltham, MA, USA. APC conjugated with Annexin V—Invitrogen™, Thermo Fisher Scientific. Dimethylsulfoxide (DMSO)—Dinâmica^®^, Indaiatuba, Brazil. Dulbecco’s modified Eagle’s medium Ham F-12 (DMEM)-Merk, Sigma-Aldrich^®^, Burlington, MA, USA, was used. Dulbecco’s phosphate-buffered saline (DPBS)—LGC Biotecnologia^®^, Cotia, Brazil. Extracts Rich in Cannabidiol (ERCBD)—NGO Maria Flor, Marília, SP, Brazil) Δ9-Tetrahydrocannabinol (ERTHC)—NGO Maria Flor, Marília, SP, Brazil. Fetal bovine serum (FBS) was obtained from Nova Biotecnologia^®^. Gentamicin—Gibco™, Thermo Fisher Scientific. Hoechst—Invitrogen™, Thermo Fisher Scientific. Propidium iodide was obtained from Invitrogen™, Thermo Fisher Scientific. Trypsin-EDTA solution—Gibco™, Thermo Fisher Scientific. 3-(4,5-dimethyl-2-thiazolyl)-2,5-diphenyl-2-H-tetrazolium bromide (MTT) Invitrogen™, Thermo Fisher Scientific.

### 2.1. Cell Lines and Cell Culture

Two canine prostate carcinoma cell lines (PC1 and PC2) were used. PC1 was established from a 10-year-old, intact mixed breed nonmetastatic dog, and PC2 was established from an 11-year-old, intact poodle dog with metastasis. The cells were previously characterized [17]. All cell lines were tested for Mycoplasma spp. Cells were grown in 75 cm^2^ culture flasks for expansion in DMEM-Ham’s F12 supplemented with 10% FBS, 1% gentamicin, and 0.5% antimycotic antibiotic solution and maintained in a humidified atmosphere of 5% CO^2^ at 37 °C.

### 2.2. Obtaining Extracts

The purities of the extracts rich in cannabidiol (ERCBD) and Δ9-tetrahydrocannabinol (ERTHC) were evaluated via HPLC by the company DALL Soluções Analíticas (Curitiba, PR, Brazil). The extracts were diluted in dimethyl sulfoxide (DMSO) at a 1:1 ratio, filtered and diluted in DMEM to reach a working dose of 100 µM CBD in the ERCBD and 100 µM THC in the ERTHC.

### 2.3. In Vitro Cytotoxicity Assay

A colorimetric MTT assay was used to determine the cytotoxic effects of ERCDB and ERTHC. For this purpose, 1 × 104 PC1 and PC2 cells/well were seeded in a 96-well plate. The doses tested were 2.5 µM, 5 µM, 7.5 µM and 10 µM for ERCBD and ERTHC, respectively.. After 24 h of seeding in DMEM supplemented with 10% FBS, the medium was replaced with DMEM without FBS supplemented with different concentrations of the test compounds under the same conditions and for the same period (24 h). The MTT assay (Invitrogen™, Thermo Fisher Scientific, USA) was performed according to the manufacturer’s instructions, and Spectro colorimetric analysis was performed in a microplate reader (570 nm range). All the cell lines were tested at four concentrations made in a serial dilution, guided by control groups, and executed in triplicate. The control groups were DMSO at the highest concentration (vehicle) of dilution and DMEM with 10% FBS.

### 2.4. Apoptosis Analysis

Cell death analysis was performed in PC1 and PC2 after 24 h of treatment with the IC50 doses of ERCBD and ERTHC. The samples were suspended in a medium containing calcium (a buffer solution) for analysis of apoptosis. To this end, 5 µL of APC-conjugated annexin V (Becton Dickinson and Company, Franklin Lakes, NJ, USA), 5 µL (1.5 µM final concentration) of propidium iodide (Becton Dickinson and Company), and 5 µL (7 µM final concentration) of Hoechst (Sigma-Aldrich^®^, USA) were added to the cell suspensions. All samples were incubated in the dark for 15 min at room temperature, and flow cytometry was performed with a final concentration of 1 × 106 cells/mL in Fortessa LSR equipment (Becton Dickinson, Mountain View, CA, USA). The filter configurations for the PMTs for measuring the fluorescence emission of the applied fluorochromes were 694/50 nm (IP), 660/20 nm (Annexin-APC), and 450/50 nm (Hoechst 33342). The acquisition rate was 30.000 events/cell line. The data were generated in a contour plot graph including the <0 t axis (biexponential), making all events visible and properly compensated through BD FACSDiva TM software v6.1 (Becton Dickinson).

### 2.5. Data Analysis

The IC50 values of all the tested compounds were obtained by plotting the spectrum colorimetric data in a dose-response curve after the application of data normalization and a nonlinear regression method in GraphPad Prism 8.0.1 software. The statistical significance (*p* < 0.05) was determined by a comparison of each tested group with the control (vehicle) group by independent t tests and ANOVA for cell viability. For the type of cell death, the number of cells was compared among the groups using ANOVA or the Kruskal-Wallis test.

## 3. Results

### 3.1. In Vitro Cytotoxicity Assay

**Figure 1:**
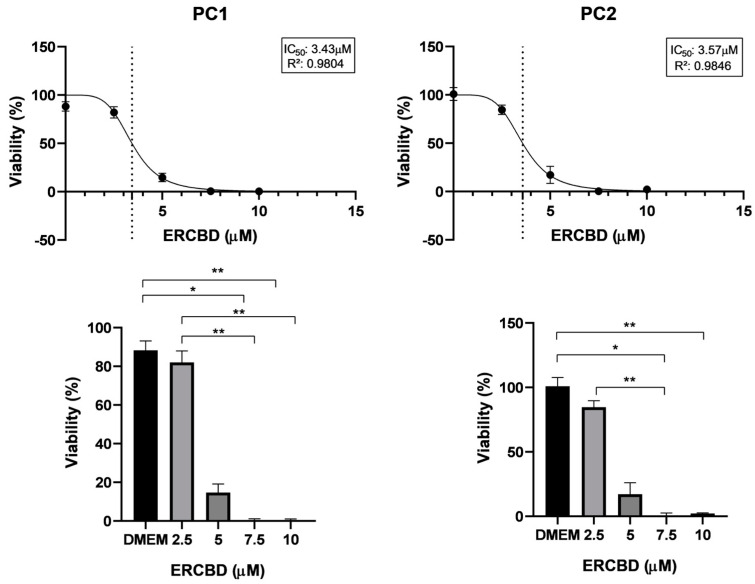
Cell viability of PC1 (canine prostate carcinoma cell line), PC2 (canine prostate carcinoma cell line) cells. Nonlinear regression curve and determination of the IC50 values after 24 h of exposure to the ERCBD (extract rich in CBD). The vertical dotted line represents the IC50 dose. The circles in the curves represent the doses used for MTT test. Student’s *t* test was used to compare two groups; ANOVA with Tukey’s comparison Kruskal-Wallis test was used to compare five groups. * *p* < 0.05; ** *p* < 0.01.

**Figure 2:**
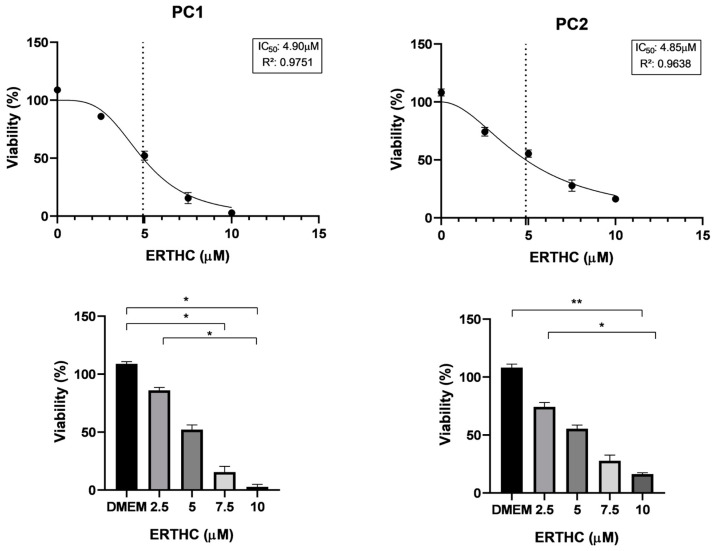
Cell viability of PC1 (canine prostate carcinoma cell line), PC2 (canine prostate carcinoma cell line) cells. Nonlinear regression curve and determination of the IC50 of doss after 24 h of exposure to ERTHC (extract rich in THC). The vertical dotted line represents the IC50 dose. The circles in the curves represent the doses used for MTT test. Student’s *t* test was used to compare two groups; ANOVA with Tukey’s comparison Kruskal-Wallis test was used to compare five groups. * *p* < 0.05; ** *p* < 0.01.

For both the canine cell lines (PC1 and PC2), there was a decrease in viability in a dose-dependent manner for the compounds ERCBD and ERTHC (Figure 1 and Figure 2).

### 3.2. Apoptosis Analysis

**Figure 3:**
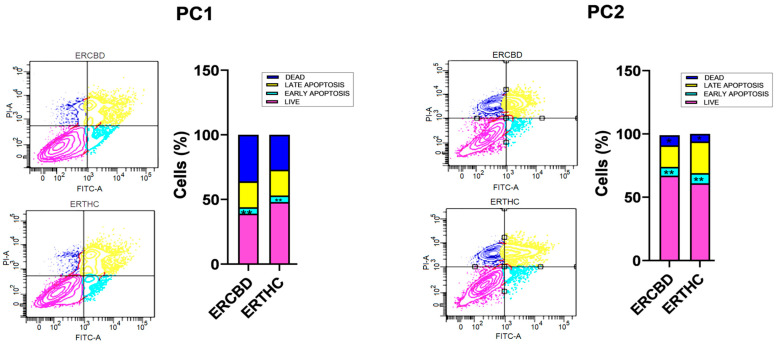
Annexin V/FITC and propidium iodide apoptosis assays of the PC1 and PC2 canine prostate carcinoma cell lines. Comparison of the IC50s (ERCBD and ERTHC), control (DMEM), and vehicle (DMSO) after 24 h of treatment with the THC-rich extract. The determination of early and late apoptosis, as well as necrosis was determined by positivity and/or negativity for Annexin V and PI. Live cells were Annexin V+ and PI+ (pink in the graph, Figure 3); dead cells were Annexin V− and PI− (dark blue); early apoptotic cells were Annexin V+ and PI− (light blue) and late apoptotic cells were Annexin V+ and PI+, according to manufacturer’s instructions. Student’s *t* test was used to compare the four groups based on the control group; ANOVA with the Kruskal-Wallis multiple comparison test was used to compare the four groups. * *p* < 0.05; ** *p* < 0.01.

The main cell death mechanism differed among the cell lines. PC1 cells were more sensitive to ERCBD and ERTHC, with 36% and 27%, respectively, of the cells dying from necrosis, followed by late apoptosis (20% of the cells for both ERCBD and ERTHC). For PC2, the main mechanism of cell death was late apoptosis (17.2% and 24.6% for the ERCBD and ERTHC groups, respectively) (Figure 3 and Table 1).

## 4. Discussion

CBD and THC-rich *Cannabis sativa* oil extracts inhibited cell proliferation in a dose-dependent manner in canine prostate carcinoma cell lines. For canine cell lines, the antitumoral effect of rich CBD and rich THC oils has not been previously reported. For both canine prostate carcinoma cell lines, IC50 values of 3.43 and 3.57 µM were found for ERCBD, and 4.90 and 4.48 µM were found for ERTHC; these concentrations are lower than those of human PC cell lines (our results and [18]. There is a large difference in the necessary IC50 to reach the anti-proliferative effect, highlighting the importance of comparative oncology to evaluate the mechanism of action of these compounds between species, as well as the need for testing for each species [18].

In an unpublished study by our group, for the human cell line (LNCaP), an IC50 of 21.49 µM was found for ERCBD, and an IC50 of 18.69 µM was found for ERTHC. In a study carried out by De Petrocellis et al. [18] in the same cell line, the IC50s were 25.0 µM for ERCBD and 16.9 µM for ERTHC; these results are very similar to those found in our study. Sarfaraz et al. [19] also obtained an IC50 of 10 µM for THC in LNCaP cells; over a period of 48 h, exposure to the drug for a longer period may explain the lower dose required compared to the IC50 found in this study as well as that reported by De Petrocellis et al. [18].

Unlike the proliferation inhibitory response, which was very similar in the two canine cell lines, the response to cell death was heterogeneous, with PC2 being more resistant to cell death than PC1. Similar results were demonstrated by Kobayashi et al. [18], as PC2 is more aggressive and less responsive than PC1 to treatment with Toceranib, a tyrosine kinase inhibitor. These results can be explained by tumor aggressiveness since the PC2 cell line is derived from an animal with metastatic PC.

One of the important points of the metastatic cascade is the epithelial–mesenchymal transition (EMT), which leads to metastatic and more aggressive tumors, and CBD has already been shown to reverse EMT by reducing IL-1β secretion, in addition to promoting intercellular junctions, increasing E-cadherin expression in tumors, in addition to silencing the IL-1β/IL-1R/β-catenin and Wnt/β-catenin pathways, both of which lead to EMT [13].

In PC, particularly in the most aggressive metastatic or advanced stage tumors, there is evidence that the intrinsic apoptosis pathways are dysfunctional [20], a decrease in cell death signaling pathways through apoptosis is one of the resistance mechanisms to drugs in tumor cells [21], and the canine prostate carcinoma lines PC1 and PC2 show upregulated MDR1 expression in comparison to a canine mammary carcinoma cell line known to be drug multidrug resistant (data not published by our group).

In the present study, we showed that CBD- and THC-rich extracts induced cell death by early and late apoptosis, in addition to necrosis, in canine prostate carcinoma cells. After treatment with cannabinoids, apoptosis can be induced via the intrinsic pathway (which is mediated by mitochondria) or by death receptor signaling on the cell surface [22]. Several studies have shown that CBD can lead to apoptosis through stress on the endoplasmic reticulum in tumor cells, through the CA2+-dependent pathway, leading to disruption of the integrity of the mitochondrial membrane [13]. As previously observed, cannabinoids affect cell death mechanisms in different human cell lines, including breast cancer, hepatocytes, and prostate cancer cells [23].

Studies have shown that cannabidiol (CBD) significantly promoted cellular apoptosis and changes in gene proteins related to cellular apoptosis in vitro in human osteosarcoma lines, and the administration of cannabidiol inhibited tumor growth and promoted apoptosis in osteosarcoma cells in a mouse xenograft model [24]. CBD reduced proangiogenic factors, including MMP2 and MMP9, endothelin-1 (ET-1), and platelet-derived growth factor-AA (PDGF-AA), which are also of utmost importance in the metastatic cascade [13].

Delta-9-tetrahydrocannabinol (THC) is in its early oncology trials and induces apoptosis in glioma cells, as determined by DNA fragmentation and loss of plasma membrane asymmetry [25]. Delta-9-THC promoted apoptosis in human prostate PC-3 cells in a dose-dependent manner [26].

ERCBD and ERTHC presented similar IC50 values between the canine cell lines used in this research, which may, in the future, facilitate the standardization of therapeutic protocols for in vitro and in vivo studies. The results of the inhibitory response and the apoptosis induction of the cells suggest that enriched extracts may provide the basis for new therapies for canine prostate carcinoma, together with currently used chemotherapeutic drugs.

## 5. Conclusions

We confirmed that ERCBD and ERTHC can inhibit cell proliferation, in addition to causing cell death through necrosis and apoptosis, in canine prostate carcinoma cell lines (PC1 and PC2), providing a promising opportunity for further studies of the in vitro and in vivo mechanisms of action of these compounds, using dogs as a natural model for prostate carcinoma clinical trials.

## Figures and Tables

**Table 1:** Percentage of dead and viable cells according to the annexin V/IP assay for canine prostatic carcinoma lines (PC1 and PC2) after treatment with an extract rich in CBD (ERCBD) and extract rich in THC (ERTHC).

	PC1		PC2	
	ERCBD	ERTHC	ERCBD	ERTHC
Early apoptose	4.4%	5.2%	7.4%	8.2%
Late apoptose	20.0%	20.0%	17.2%	24.6%
Dead	36.0%	27.0%	8.0%	6.0%
Live	39.6%	47.80%	67.4%	61.2%

## Data Availability

The datasets generated during and/or analyzed during the current study are available from the corresponding author upon reasonable request.

## References

[1] Rizzo A., Santoni M., Mollica V., Fiorentino M., Brandi G., Massari F. (2022). Microbiota and prostate cancer. Semin. Cancer Biol..

[2] Sekhoacha M., Riet K., Motloung P., Gumenku L., Adegoke A., Mashele S. (2022). Prostate Cancer Review: Genetics, Diagnosis, Treatment Options, and Alternative Approaches. Molecules.

[3] Wasim S., Lee S.-Y., Kim J. (2022). Complexities of Prostate Cancer. Int. J. Mol. Sci..

[4] Leroy B.R., Northrup N. (2009). Prostate cancer in dogs: Comparative and clinical aspects. Vet. J..

[5] Nascimento-Gonçalves E., Seixas F., Ferreira R., Oliveira P.A., Colaço B. (2023). In vivo prostate cancer research: Key interspecies prostate anatomical features for translational medicine. Open Vet. J..

[6] Nascente E.d.P., Amorim R.L., Fonseca-Alves C.E., de Moura V.M.B.D. (2022). Comparative Pathobiology of Canine and Human Prostate Cancer: State of the Art and Future Directions. Cancers.

[7] Stans J. (2020). Prostatectomy as a treatment for canine prostate cancer: A literature review. Open Vet. J..

[8] Laufer-Amorim R., Fonseca-Alves C.E., Villacis R.A.R., Linde S.A.D., Carvalho M., Larsen S.J., Marchi F.A., Rogatto S.R. (2019). Comprehensive Genomic Profiling of Androgen-Receptor-Negative Canine Prostate Cancer. Int. J. Mol. Sci..

[9] Argyle D.J. (2009). Prostate cancer in dogs and men: A unique opportunity to study the disease. Veter J..

[10] Rock E.M., Parker L.A. (2021). Constituents of Cannabis Sativa. Adv. Exp. Med. Biol..

[11] Huang T., Xu T., Wang Y., Zhou Y., Yu D., Wang Z., He L., Chen Z., Zhang Y., Davidson D. (2021). Cannabidiol inhibits human glioma by induction of lethal mitophagy through activating TRPV4. Autophagy.

[12] McAllister S.D., Soroceanu L., Desprez P.-Y. (2015). The Antitumor Activity of Plant-Derived Non-Psychoactive Cannabinoids. J. Neuroimmune Pharmacol..

[13] Yan C., Li Y., Liu H., Chen D., Wu J. (2023). Antitumor mechanism of cannabidiol hidden behind cancer hallmarks. Biochim. et Biophys. Acta (BBA) Rev. Cancer.

[14] Massi P., Solinas M., Cinquina V., Parolaro D. (2013). Cannabidiol as potential anticancer drug. Br. J. Clin. Pharmacol..

[15] Gross C., Ramirez D.A., McGrath S., Gustafson D.L. (2021). Cannabidiol Induces Apoptosis and Perturbs Mitochondrial Function in Human and Canine Glioma Cells. Front. Pharmacol..

[16] Pondugula S.R., Boothe D. (2024). Effects of Cannabidiol, ∆9-Tetrahydrocannabinol, and WIN 55-212-22 on the Viability of Canine and Human Non-Hodgkin Lymphoma Cell Lines. Biomolecules.

[17] Kobayashi P.E., Lainetti P.F., Leis-Filho A.F., Delella F.K., Carvalho M., Cury S.S., Carvalho R.F., Fonseca-Alves C.E., Laufer-Amorim R. (2020). Transcriptome of Two Canine Prostate Cancer Cells Treated with Toceranib Phosphate Reveals Distinct Antitumor Profiles Associated with the PDGFR Pathway. Front. Veter Sci..

[18] De Petrocellis L., Ligresti A., Schiano Moriello A., Iappelli M., Verde R., Stott C.G., Cristino L., Orlando P., Di Marzo V. (2013). Non-THC cannabinoids inhibit prostate carcinoma growth in vitro and in vivo: Pro-apoptotic effects and underlying mecha-nisms. Br. J. Pharmacol..

[19] Sarfaraz S., Afaq F., Adhami V.M., Mukhtar H. (2005). Cannabinoid receptor as a novel target for the treatment of prostate cancer. Cancer Res..

[20] Campbell K.J., Leung H.Y. (2021). Evasion of cell death: A contributory factor in prostate cancer development and treatment resistance. Cancer Lett..

[21] Todaro M., Lombardo Y., Francipane M.G., Alea M.P., Cammareri P., Iovino F., Di Stefano A.B., Di Bernardo C., Agrusa A., Condorelli G. (2008). Apoptosis resistance in epithelial tumors is mediated by tumor-cell-derived inter-leukin-4. Cell Death Differ..

[22] Shrivastava A., Kuzontkoski P.M., Groopman J.E., Prasad A. (2011). Cannabidiol Induces Programmed Cell Death in Breast Cancer Cells by Coordinating the Cross-talk between Apoptosis and Autophagy. Mol. Cancer Ther..

[23] Guzmán M. (2003). Cannabinoids: Potential anticancer agents. Nat. Rev. Cancer.

[24] Xu F., Sun G., Peng Z., Liu J., Li Z., Yan J. (2022). Cannabidiol promotes apoptosis of osteosarcoma cells in vitro and in vivo by activating the SP1-CBX2 axis. Am. J. Transl. Res..

[25] Sánchez C., Galve-Roperh I., Canova C., Brachet P., Guzmán M. (1998). Delta9-tetrahydrocannabinol induces apoptosis in C6 glioma cells. FEBS Lett..

[26] Ruiz: L., Miguel A., Díaz-Laviada I. (1999). Δ^9^-Tetrahydrocannabinol induces apoptosis in human prostate PC-3 cells via a receptor-independent mechanism. FEBS Lett..

